# The Chloroplast SRP Systems of *Chaetosphaeridium globosum* and *Physcomitrella patens* as Intermediates in the Evolution of SRP-Dependent Protein Transport in Higher Plants

**DOI:** 10.1371/journal.pone.0166818

**Published:** 2016-11-18

**Authors:** Dominik Ziehe, Beatrix Dünschede, Mira Zenker, Silke Funke, Marc M. Nowaczyk, Danja Schünemann

**Affiliations:** 1 Molecular Biology of Plant Organelles, Ruhr-University Bochum, 44780, Bochum, Germany; 2 Cyanobacterial Membrane Protein Complexes, Ruhr-University Bochum, 44780, Bochum, Germany; Istituto di Genetica Molecolare, ITALY

## Abstract

The bacterial signal recognition particle (SRP) mediates the cotranslational targeting of membrane proteins and is a high affinity complex consisting of a SRP54 protein subunit (Ffh) and an SRP RNA. The chloroplast SRP (cpSRP) pathway has adapted throughout evolution to enable the posttranslational targeting of the light harvesting chlorophyll a/b binding proteins (LHCPs) to the thylakoid membrane. In spermatophytes (seed plants), the cpSRP lacks the SRP RNA and is instead formed by a high affinity interaction of the conserved 54-kD subunit (cpSRP54) with the chloroplast-specific cpSRP43 protein. This heterodimeric cpSRP recognizes LHCP and delivers it to the thylakoid membrane. However, in contrast to spermatophytes, plastid SRP RNAs were identified within all streptophyte lineages and in all chlorophyte branches. Furthermore, it was shown that cpSRP43 does not interact with cpSRP54 in chlorophytes (e.g., *Chlamydomonas reinhardtii*). In this study, we biochemically characterized the cpSRP system of the charophyte *Chaetosphaeridium globosum* and the bryophyte *Physcomitrella patens*. Interaction studies demonstrate low affinity binding of cpSRP54 to cpSRP43 (K_d_ ~10 μM) in *Chaetosphaeridium globosum* and *Physcomitrella patens* as well as relatively low affinity binding of cpSRP54 to cpSRP RNA (K_d_ ~1 μM) in *Physcomitrella patens*. CpSRP54/cpSRP43 complex formation in charophytes is supported by the finding that specific alterations in the second chromodomain of cpSRP43, that are conserved within charophytes and absent in land plants, do not interfere with cpSRP54 binding. Furthermore, our data show that the elongated apical loop structure of the *Physcomitrella patens* cpSRP RNA contributes to the low binding affinity between cpSRP54 and the cpSRP RNA.

## Introduction

**Fig 1:**
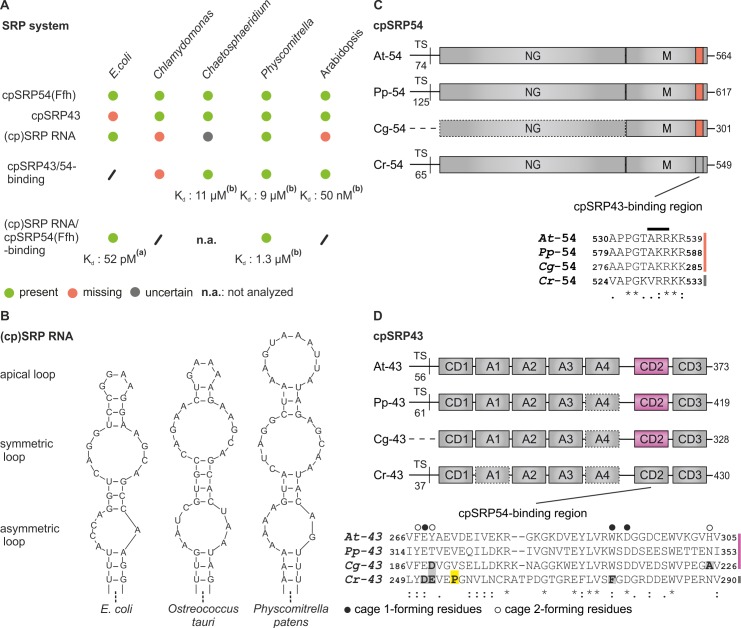
CpSRP components in various organisms of the green lineage. (A) The presence of a SRP54 subunit, cpSRP43 and cpSRP RNA in the indicated SRP systems is denoted by green dots, while the lack of a component is marked with a red dot. In addition, the ability or inability of (cp)SRP54 to bind one of these components is also represented by a green or red dot, respectively. The given K_d_ values were either described previously in Buskiewicz et al., 2005^(a)^ [22] or were obtained in this work ^(b)^. (B) Comparison of different (cp)SRP RNA structures of *E*. *coli*, *Ostreococcus tauri* and *Physcomitrella patens*. All (cp)SRP RNAs are composed of an asymmetric loop, a symmetric loop and an apical loop region. *E*. *coli* and *Ostreococcus tauri* (cp)SRP RNA harbor a conserved tetraloop structure while this structure is elongated in *Physcomitrella patens* cpSRP RNA. (C) The cpSRP54 protein consists of an N-terminal NG-domain with GTPase activity and a C-terminal M-domain where the cpSRP43 binding motif is localized. *Arabidopsis thaliana* (At), *Physcomitrella patens* (Pp) and *Chaetosphaeridium globosum* (Cg) cpSRP54 harbor the conserved A(R/K)R cpSRP43-binding motif (see red boxes and alignment). The numbering of the amino acid sequence of Cg-54M refers to the used EST clone as described in materials and methods. *Chlamydomonas reinhardtii* cpSRP54 is not complexed with cpSRP43 because of a valine instead of an alanine in the cpSRP43-binding motif (see grey box and alignment). (D) CpSRP43 is composed of three chromodomains (CD1-CD3) and four ankyrin repeats (A1-A4). The cpSRP54 binding region is located in chromodomain 2 (CD2). CD2 of *Arabidopsis thaliana* (At), *Physcomitrella patens* (Pp) and *Chaetosphaeridium globosum* (Cg) forms two aromatic cages that recognize the A(R/K)R cpSRP43-binding motif (see purple boxes and alignment) [19]. Residues forming the cage 1 and cage 2 regions in *Arabidopsis thaliana* cpSRP43 and the corresponding positions in *Physcomitrella patens*, *Chaetosphaeridium globosum* and *Chlamydomonas reinhardtii* cpSRP43 are highlighted with black and white circles. Residues in *Chaetosphaeridium globosum* and *Chlamydomonas reinhardtii* cpSRP43, which differ in these positions from *Arabidopsis thaliana* and *Physcomitrella patens* cpSRP43, are marked by gray boxes. Pro-255 in *Chlamydomonas reinhardtii* cpSRP43 that interferes with cpSRP54 binding is marked by a yellow box. Symbols display the degree of conservation: identical residues (asterisk), conserved substitution (colon), and semiconserved substitution (dot); TS, transit sequence.

The light harvesting chlorophyll a/b binding proteins (LHCP) form the light harvesting antennas of photosystems I and II and are the most abundant thylakoid membrane proteins [1]. The nuclear encoded LHCPs are first imported into the chloroplast and subsequently posttranslationally inserted into the thylakoid membrane. The transfer of the hydrophobic LHCPs through the stromal compartment and the insertion into the thylakoid membrane is mediated by the posttranslational chloroplast signal recognition particle (cpSRP) pathway [2, 3]. The cpSRP pathway originates in the bacterial SRP system, which mediates the cotranslational targeting of most membrane proteins to the bacterial plasma membrane [4, 5]. Here, the SRP recognizes nascent chains of these proteins and guides the ribosome-nascent chain complex to the Sec translocon by interacting with the SRP receptor FtsY. Bacterial SRP consists of two essential components, a homolog of the universally conserved SRP54 protein (Ffh) and an SRP RNA [6], which bind with picomolar affinity (Fig 1A). The posttranslational cpSRP pathway has been best studied in the spermatophyte (seed plant) *Arabidopsis thaliana*. It was shown that this system combines ancestral bacterial with novel chloroplast-specific components. CpSRP contains the conserved SRP54 subunit (cpSRP54) but differs from bacterial SRP because it forms a high affinity complex with the novel component cpSRP43 and lacks an SRP RNA ([7–9], Fig 1A). The heterodimeric cpSRP binds the imported LHCP to form the soluble transit complex, which is recruited to the thylakoid membrane by interacting with the chloroplast SRP receptor cpFtsY and the Alb3 translocase [10–16]. The cpSRP subunits, cpSRP54 and cpSRP43, are phylogenetically conserved in the green plant lineage including all land plants as well as the two distinct lineages of green algae, charophytes and chlorophytes [9]. Notably, the absence of an SRP RNA component in chloroplasts is only typical for spermatophytes. CpSRP RNAs were identified within all nonspermatophyte land plant lineages and in all green algae branches [9]. However, the structure of the chloroplast SRP RNAs is much more diverse than that of bacterial SRP RNAs. The moss *Physcomitrella patens*, for example, contains a cpSRP RNA with an elongated apical loop instead of the classical tetraloop, which is typically present in bacterial SRP RNAs [9, 17] (Fig 1B). Interestingly, a recent study demonstrated that the cpSRP system in chlorophytes (e.g., *Chlamydomonas reinhardtii*) differs from that of land plants in that cpSRP43 is not complexed to cpSRP54 ([15], Fig 1A). The inability of the cpSRP complex formation is due to alterations within the cpSRP54 C-terminal tail region and the second chromodomain (CD2) of cpSRP43, which form the binding interface in *Arabidopsis thaliana*. Here, CD2 forms two aromatic cages that are crucial for recognizing the cpSRP43-binding motif ARR that is located in close proximity to the C-terminus of cpSRP54 [15, 18, 19] (Fig 1C and 1D). The cpSRP54 tail of chlorophytes (e.g., *Chlamydomonas reinhardtii*) does not contain the within streptophytes conserved cpSRP43-binding motif A(R/K)R but displays a valine instead of an alanine, which interferes with cpSRP43 binding [15] (Fig 1C). The cpSRP43 proteins of chlorophytes differ from cpSRP43 of land plants by displaying a proline in the first β-strand of CD2, which is detrimental to cpSRP54-binding ([15], Fig 1D). The inability of cpSRP54 to bind cpSRP43 in chlorophytes has a significant impact on its function because the involvement of cpSRP54 in transit complex formation is dependent on the interaction with cpSRP43 [15]. In contrast with chlorophytes, cpSRP54 proteins of charophytes (e.g., *Chaetosphaeridium globosum*), the immediate green algal ancestors of land plants, harbor the canonical cpSRP43-binding motif (Fig 1C). Additionally, their cpSRP43 proteins are similar to land plant cpSRP43 proteins in that they do not possess a proline in the first β-strand of CD2 and the three residues forming the aromatic cage 1 in *Arabidopsis thaliana* are conserved ([15], Fig 1D). However, the aromatic cage 2 of charophyte cpSRP43 differs from *Arabidopsis thaliana*. Instead of an aromatic tyrosine, which is important for recognizing the ARR binding motif in *Arabidopsis thaliana* cpSRP54, charophyte cpSRP43 proteins exhibit a negatively charged residue. ([15, 19], Fig 1D).

This study aimed to answer the question of whether cpSRP complex formation occurs in charophytes. Furthermore, binding affinities for cpSRP54/cpSRP43 and cpSRP54/(cp)SRP RNA interactions in various branches of the green lineage were determined and compared with the (cp)SRP system of *Arabidopsis thaliana* or bacteria.

## Materials and Methods

### Plasmids and plasmid construction

The cDNAs coding for *Chaetosphaeridium globosum* cpSRP54M (Cg-cpSRP54M) and cpSRP43 (Cg-cpSRP43) were synthesized by GenArt® Life Technologies (Thermo Scientific) according to Träger et al., 2012 [9]. The EST clone with the accession HO382660 encodes a partial sequence of Cg-cpSRP54, which corresponds to the C-terminal M-domain and the EST clone with the accession HO370201 encodes Cg-cpSRP43. The amino acid sequence of Cg-cpSRP54M and Cg-cpSRP43 is indicated in S1 Table.

For yeast two-hybrid experiments, the coding sequence for mature *Physcomitrella patens* cpSRP43 (Pp-cpSRP43) was cloned into the pACT2 plasmid using NcoI/EcoRI restriction sites. The coding sequences for mature *Arabidopsis thaliana* cpSRP43 (At-cpSRP43), At-cpSRP54 and Pp-cpSRP54 were cloned into pACT2 and pGBKT7 as described [9, 18, 20]. Mutation constructs pACT2-At-cpSRP43(Y269D), pACT2-At-cpSRP43(Y269E), pACT2-Pp-cpSRP43(T317D) and pACT2-Pp-cpSRP43(T317E) were obtained using site directed mutagenesis PCR (Agilent Technologies) with the initially described plasmids as a template.

For the overexpression of Pp-GST-cpSRP43 and Cg-GST-cpSRP43, the corresponding coding sequences were cloned into the pGEX4T3 plasmid (GE Healthcare) using BamHI/SalI restriction sites. pGEX4T3-At-GST-cpSRP43 was described previously [7]. Mutation constructs pGEX4T3-At-GST-cpSRP43(Y269D), pGEX4T3-At-GST-cpSRP43(Y269E), pGEX4T3-Pp-GST-cpSRP43(T317D), pGEX4T3-Pp-GST-cpSRP43(T317E) and pGEX4T3-Cg-GST-cpSRP43(V192P) were generated using mutagenesis PCR with the above described plasmids as a template. To obtain Cg-His-cpSRP54M and Cg-His-cpSRP43, the corresponding coding sequences were cloned into pET^TM^Duet-1 (*Merck Bioscience)* by the restriction enzymes BamHI/SalI. The mutated Cg-His-cpSRP43(V192P) was generated using site directed mutagenesis PCR with the originally described plasmid as a template. The overexpression constructs encoding At-His-cpSRP54, At-His-cpSRP54M, Pp-cpSRP54-His, Pp-His-cpSRP43 as well as Pp-His-cpSRP43ΔCD1 were described previously [7, 9, 12, 18]. To generate a Cg-His-eGFP-cpSRP43 fusion construct, the sequence of eGFP was cloned into pET^TM^Duet-1 using the restriction enzymes BamHI/SalI. Subsequently, the amplified DNA of Cg-cpSRP43 was cloned into the EcoRI/SalI site of the prepared pET^TM^Duet-1 -eGFP vector.

For *in vitro* transcription, the DNA coding for *Physcomitrella patens* cpSRP RNA, *Ostreococcus tauri* cpSRP RNA, *E*. *coli* SRP RNA and *Physcomitrella patens/E*. *coli* (Pp/Ec)-hybrid SRP RNA was synthesized and cloned into a pUC-57 plasmid (GenScript) as described previously [9]. In the hybrid SRP RNA, the 10 bp elongated apical loop (AAGUAAAUUA) of the *Physcomitrella patens* SRP RNA was replaced with the tetraloop (GGAA) of *E*. *coli* SRP RNA.

### Expression and purification of proteins

His-tag fusion constructs were expressed in *E*. *coli* strains BL21(DE3) or Rosetta2(DE3) (*Merck Bioscience*). Bacteria cells were cultivated in LB medium at 37°C to an optical density between 0.6 and 0.8. After induction with 1 mM of isopropyl-β-D-thiogalactopyranosid (IPTG) the cells were grown for additional 3 hours at 37°C. Cells were collected by centrifugation (10 min, 4500 x g, 4°C) and resuspended in washing buffer (25 mM HEPES, 200 mM NaCl, 20 mM imidazole, 5 mM MgCl_2_, 2 mM DTT, pH 8.0). Cells were disrupted by sonification and after centrifugation the supernatant was either loaded onto a nickel-nitrilotriacetic acid column (GE Healthcare) or incubated with nickel-nitrilotriacetic acid resin (Qiagen). After washing His-tag fusion constructs were eluted with elution buffer (25 mM HEPES, 200 mM NaCl, 250 mM imidazole, 5 mM MgCl_2_, 2 mM DTT, pH 8.0) and desalted using PD-10 columns (GE Healthcare) or the ÄKTA-purifier system with Superdex200 10/300 GL columns (GE Healthcare) and eluted in thermophoresis or column buffer as described below. GST-fusion constructs were expressed in *E*. *coli* strains BL21 (DE3) or Rosetta2(DE3) (*Merck Bioscience*). *E*. *coli* cells were grown as described above with the following modifications. After induction with 1 mM of isopropyl-β-D-thiogalactopyranosid (IPTG) the cells were grown for 1 hour at 28°C. Proteins were purified using glutathione-sepharose (GE Healthcare), washed with PBS buffer (300 mM NaCl, 2.7 mM KCl, 10 mM Na_2_HPO_4_, 1.8 mM K_2_HPO_4_, 20 mM imidazole, 2 mM DTT, pH 7.3) and eluted with 10 mM reduced glutathione in PBS buffer. For further experiments, GST-fusion constructs were desalted using PD-10 columns (GE Healthcare) and eluted in PBS buffer.

### In vitro transcription

*In vitro* transcription of the indicated SRP RNAs was performed as previously described [9] using the TranscriptAid T7 high-yield transcription kit (Fermentas) according to the manufacturer´s instructions.

### Yeast two-hybrid analysis

The yeast two-hybrid assays were performed as described previously [18]. The co-transformed yeast Y190 cells were dotted onto minimal media lacking Leu and Trp (-LT) and Leu, Trp and His (-LTH).

### Microscale thermophoresis

The indicated proteins (Pp-His-cpSRP43, Pp-cpSRP54-His, and At-His-cpSRP43) were labeled using the Monolith™ NT.115 Protein Labeling Kit RED-NHS (Amine Reactive) according to the manufacturer's instructions. For further experiments, an eGFP-fusion construct of Cg-cpSRP43 was used to prevent sticking to the capillary walls. Protein-protein and protein-RNA interaction studies were analyzed in thermophoresis buffer (300 mM NaCl, 2.7 mM KCl, 10 mM Na_2_HPO_4_, 1.8 mM K_2_HPO_4_, 5 mM MgCl_2_, 2 mM DTT, 0.05% Tween20, pH 7.3) by using Monolith™ NT.115 MST Premium Coated Capillaries or Hydrophobic Capillaries. The measurements were performed at MST power 20% and LED power 20% using the Monolith NT.115 instrument (NanoTemper Technologies GmbH, Munich, Germany). Thus, a dilution series of the indicated proteins or RNAs in a micromolar range was created, while the concentration of labeled ligand (Pp-His-cpSRP43, Pp-cpSRP54-His, Ath-His-cpSRP43, Cg-His-eGFP-43) was kept constant in a nanomolar range. All experiments were performed at least twice and were evaluated using the MO.Affinity Analysis Software (NanoTemper Technologies GmbH, Munich, Germany).

### Pull-down analysis

*In vitro* pull-down analyses were performed using 20 μg of the indicated GST- and His-fusion constructs according to Dünschede et al., 2015 [15].

### Size-exclusion chromatography (SEC)

Equimolar amounts of *Chaetosphaeridium globosum* His-tag fusion constructs were incubated in column buffer (20 mM HEPES NaOH, pH 8.0, 300 mM NaCl) and loaded onto a Superdex 200 10/300 GL column (GE Healthcare). Gel filtration runs were performed in column buffer at a flow rate of 0.4 ml/min. Selected fractions were then analyzed by SDS-PAGE and Coomassie staining. For further experiments, equimolar amounts of *Physcomitrella patens* His-tag fusion constructs or cpSRP RNA were mixed and incubated for 30 min at 4°C in column buffer (25 mM HEPES NaOH, pH 8.0, 300 mM NaCl, 5 mM MgCl_2_, 5% (v/v) glycerol and 2 mM DTT) before loading onto a Superdex200 10/300 GL column (GE Healthcare). Gel filtration analysis was performed in column buffer at a flow rate of 0.4 ml/min and analyzed as described.

## Results

### CpSRP complex formation is not abolished by a negatively charged residue in cage 2 of CD2 of cpSRP43

**Fig 2:**
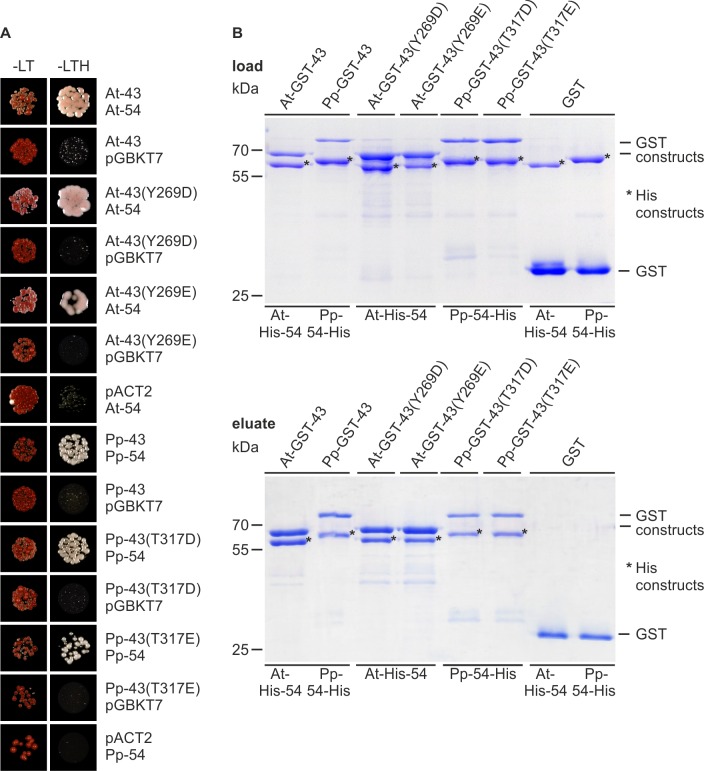
Interaction analysis between cpSRP54 and various cpSRP43 constructs of *Arabidopsis thaliana* and *Physcomitrella patens*. (A) Yeast two-hybrid interaction studies. For yeast two-hybrid assays, the yeast strain Y190 was co-transformed with pGBKT7 constructs encoding full-length cpSRP54 (54) and pACT2 constructs encoding cpSRP43 (43) or the indicated cpSRP43 mutants of *Arabidopsis thaliana* (At) and *Physcomitrella patens* (Pp). Co-transformed cells were dotted onto minimal media lacking Leu and Trp (-LT) to check for co-transformation, or lacking Leu, Trp and His (-LTH) to assess interaction. Negative controls were conducted with an empty vector (pGBKT7 or pACT2). (B) *In vitro* pull-down assays were performed with recombinant GST-cpSRP43 (At-, Pp-GST-43) constructs and His-tagged cpSRP54 proteins (At-His-54, Pp-54-His) as indicated, using glutathione-sepharose. Control reactions were performed with recombinant GST. One-tenth of the loaded proteins (upper panel) and one-third of eluted proteins (lower panel) were separated using SDS-PAGE and detected using Coomassie staining. The asterisk (*) indicates the used His-tagged constructs.

The cpSRP43 proteins of charophytes exhibit a negatively charged residue at the second position in aromatic cage 2 of CD2 instead of tyrosine in *Arabidopsis thaliana* or threonine in *Physcomitrella patens* or other land plants (Fig 1D and [15]). To analyze the general influence of this change in CD2 on cpSRP54 binding, tyrosine at position 269 in *Arabidopsis thaliana* cpSRP43 (At-cpSRP43) and the corresponding threonine at position 317 in *Physcomitrella patens* cpSRP43 (Pp-cpSRP43) (Fig 1D) were changed into aspartate or glutamate, resulting in At-cpSRP43(Y269D) or At-cpSRP43(Y269E) and Pp-cpSRP43(T317D) or Pp-cpSRP43(T317E). Binding of these cpSRP43 constructs to cpSRP54 of *Arabidopsis thaliana* or *Physcomitrella patens* (At-cpSRP54 or Pp-cpSRP54) was tested in yeast two-hybrid experiments including empty vector controls. As shown in Fig 2A, clear interactions were detected of the wildtype and mutant cpSRP43 constructs with cpSRP54 from *Arabidopsis thaliana* and *Physcomitrella patens*. To confirm the ability of the mutant cpSRP43 constructs to interact with cpSRP54, *in vitro* pull-down experiments were performed using recombinant GST-cpSRP43 constructs (At-GST-cpSRP43, At-GST-cpSRP43(Y269D), At-GST-cpSRP43(Y269E), Pp-GST-cpSRP43, Pp-cpSRP43(T317D) and Pp-cpSRP43(T317E)) and His-tagged cpSRP54 from *Arabidopsis thaliana* and *Physcomitrella patens*. Negative control reactions were conducted with recombinant GST (Fig 2B). All GST-cpSRP43 constructs clearly coprecipitated the corresponding His-tagged cpSRP54 proteins, confirming the yeast two-hybrid results. Together, these data demonstrate that a negatively charged residue at the second position in aromatic cage 2 of CD2 (corresponding to the position of Y269 in At-cpSRP43) is not detrimental to cpSRP complex formation.

### CpSRP complex formation in the charophyte *Chaetosphaeridium globosum*

**Fig 3:**
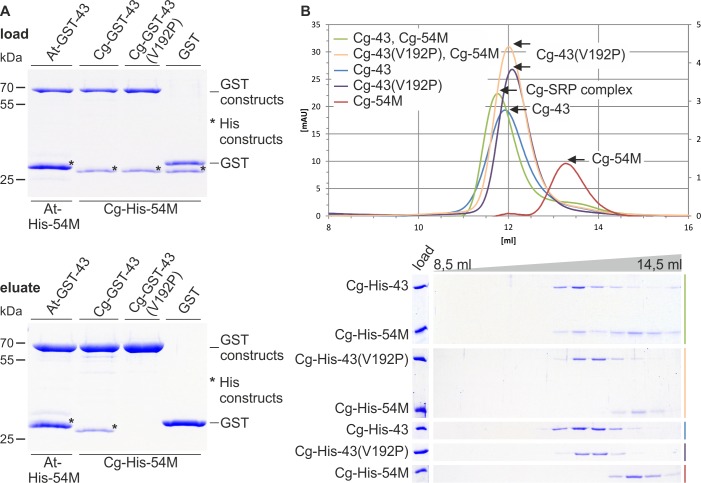
Interaction analysis between cpSRP54M and various cpSRP43 constructs of *Chaetosphaeridium globosum*. (A) *In vitro* pull-down assays were performed as described previously [15]. Combinations of recombinant GST-cpSRP43 (At-, Cg-GST-43) or the mutant construct Cg-GST-43(V192P) and His-tagged cpSRP54M (At-, Cg-His-54M) proteins were analyzed as indicated using glutathione-sepharose. Control reactions were performed with recombinant GST. One-tenth of the loaded proteins (upper panel) and one-third of eluted proteins (lower panel) were analyzed by SDS-PAGE and Coomassie staining. The asterisk (*) indicates the used His-tagged constructs. (B) Protein-protein interactions between His-tagged *Chaetosphaeridium globosum* cpSRP54M (Cg-54M) and cpSRP43 (Cg-43) or cpSRP43(V192P) were analyzed by size exclusion chromatography using equimolar amounts of the indicated recombinant proteins: (green) Cg-His-43 and Cg-His-54M, (orange) Cg-His-43(V192P) and Cg-His-54M, (blue) Cg-His-43, (violet) Cg-His-43(V192P), and (red) Cg-His-54M. Elution fractions in a range from 8.5 to 14.5 ml were separated by SDS-PAGE and detected by Coomassie staining.

To analyze cpSRP complex formation in charophytes, the binding between the cpSRP subunits of the charophyte *Chaetosphaeridium globosum* was tested. Therefore, *in vitro* pull-down experiments were conducted using recombinant GST-cpSRP43 and the His-tagged M-domain of cpSRP54 (His-cpSRP54M) from *Chaetosphaeridium globosum*. A positive control reaction contained the recombinant GST-cpSRP43 and His-cpSRP54M from *Arabidopsis thaliana* and a negative control was conducted using recombinant GST (Fig 3A). As an additional negative control, a *Chaetosphaeridium globosum* cpSRP43 construct containing a proline instead of a valine in the first β-strand of CD2 was generated (GST-cpSRP43(V192P) (Fig 1D and Fig 3A). The detrimental effect of a proline in this position on binding cpSRP54 was previously described [15]. As shown in Fig 3A, *Chaetosphaeridium globosum* GST-cpSRP43 coprecipitated *Chaetosphaeridium globosum* cpSRP54M, while no interaction was observed using GST-cpSRP43(V192P) or GST alone. Because the *Chaetosphaeridium globosum* cpSRP proteins were not stably expressed in yeast cells (data not shown), gel filtration chromatography using recombinant proteins was performed to confirm the cpSRP complex formation in *Chaetosphaeridium globosum*. When a combination of GST-cpSRP43 and His-cpSRP54M was injected onto a gel filtration column, a clear cofractionation of these proteins was observed, while the single proteins were eluted in separate fractions. As expected, a separate elution of the proteins was also observed for the combination of His-cpSRP54M and the mutant construct GST-cpSRP43(V192P) (Fig 3B).

### The binding affinity of the cpSRP43/cpSRP54 complex in *Arabidopsis thaliana* is about 200-fold higher than in *Physcomitrella patens* and *Chaetosphaeridium globosum*

**Fig 4:**
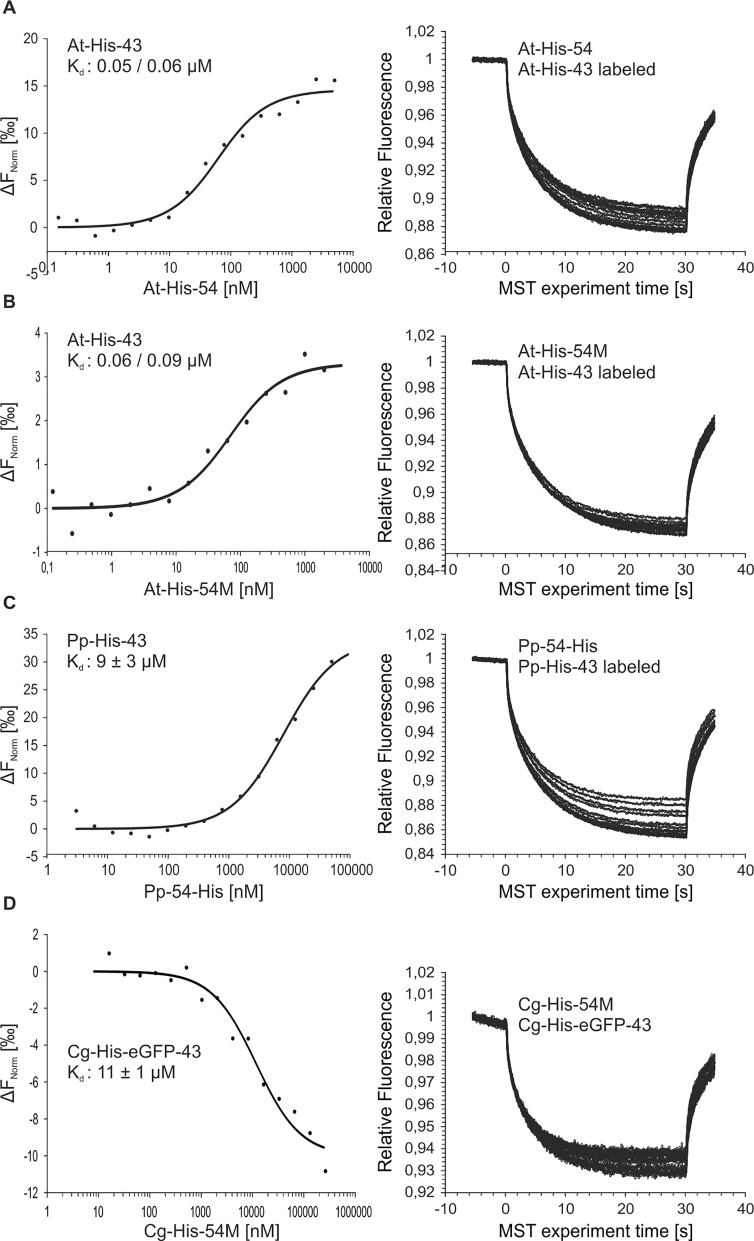
Determination of binding affinities of cpSRP54/cpSRP43 complex formation from *Arabidopsis thaliana*, *Physcomitrella patens* and *Chaetosphaeridium globosum* using microscale thermophoresis. Fluorescently labeled cpSRP43 (At-43 or Pp-43) or eGFP-Cg-43 was kept constant at 20 nM or 75 nM, respectively. The indicated cpSRP54 constructs were titrated in a micromolar excess (e.g., up to 5 μM (At-54), 100 μM (Pp-54) and 265 μM (Cg-54M)) ((A/B) *Arabidopsis thaliana*, (C) *Physcomitrella patens*, (D) *Chaetosphaeridium globosum*). The difference in normalized fluorescence [‰] was plotted against the concentration of the indicated cpSRP54 constructs (left panel) (right panel, raw MST-traces). For cpSRP complex formation analysis in *Chaetosphaeridium globosum*, the M-domain of cpSRP54 (cpSRP54M) was used. All experiments were performed at least twice, and the binding affinity (K_d_) was evaluated using the MO.Affinity Analysis Software (NanoTemper Technologies GmbH, Munich, Germany).

To further characterize the cpSRP complex formation within organisms of the green lineage, the binding affinities of the recombinant cpSRP subunits from *Arabidopsis thaliana*, *Physcomitrella patens* and *Chaetosphaeridium globosum* were assessed using microscale thermophoresis. In the experiments, fluorescently labeled His-tagged cpSRP43 from *Arabidopsis thaliana* and *Physcomitrella patens* and His-GFP-tagged cpSRP43 from *Chaetosphaeridium globosum* were mixed with increasing amounts of His-tagged cpSRP54 from *Arabidopsis thaliana* and *Physcomitrella patens* or His-cpSRP54M from *Arabidopsis thaliana* and *Chaetosphaeridium globosum*, and the thermophoretic movement of the fluorescent cpSRP43 was monitored (Fig 4A–4D and S1A–S1D Fig). A dissociation constant (K_d_) in the low nanomolar range (0.05 μM / 0.06 μM) was observed for the interaction of the full-length *Arabidopsis thaliana* cpSRP subunits (Fig 4A), and a K_d_ value in the same range (0.06 μM / 0.09 μM) was measured when the experiment was conducted with the His-tagged M-domain of cpSRP54 (Fig 4B and S1B Fig). These data support a previous observation of a high affinity interaction in the low nanomolar range using *Arabidopsis thaliana* cpSRP43 and cpSRP54M [21]. Notably, significantly higher K_d_ values of 9 ± 3 μM and 11 ± 1 μM were detected for the binding between the cpSRP43 and cpSRP54 subunits of *Physcomitrella patens* and between cpSRP43 and cpSRP54M of *Chaetosphaeridium globosum* (Fig 4C and 4D and S1C and S1D Fig). These data demonstrate that the binding between cpSRP43 and cpSRP54 in *Physcomitrella patens* and *Chaetosphaeridium globosum* exhibits an approximately 160- to 200-fold weaker affinity than in *Arabidopsis thaliana*.

### The cpSRP system in chloroplasts of *Physcomitrella patens* exhibits a binding affinity between the SRP54 protein and the SRP RNA component of about 1 μM

**Fig 5:**
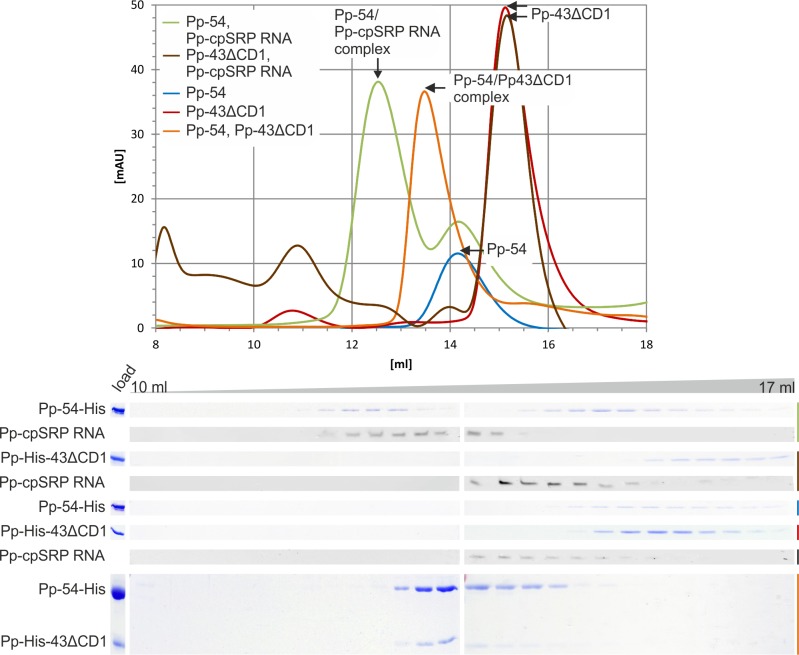
Analysis of complex formation between cpSRP54, cpSRP RNA and cpSRP43 from *Physcomitrella patens* by gel filtration. Complex formation of *Physcomitrella patens* cpSRP54 (Pp-54) and a truncated cpSRP43 construct (Pp-43ΔCD1) as well as Pp-cpSRP RNA were analyzed using size exclusion chromatography with equimolar amounts of the indicated components: (green) Pp-54-His and Pp-cpSRP RNA, (brown) Pp-His-43ΔCD1 and Pp-cpSRP RNA, (blue) Pp-54-His, (red) Pp-His-43ΔCD1, (black, not depicted in the chromatogram) Pp-cpSRP RNA, (orange) Pp-54-His and Pp-His-43ΔCD1. Elution fractions in a range from 10 to 17 ml were separated using SDS-PAGE and detected using Coomassie staining. Elution fractions containing RNA were analyzed using polyacrylamide gels and detected using SYBR Safe DNA gel stain.

The cpSRP RNA from *Physcomitrella patens* shows a similar structure to bacterial SRP RNAs but differs from classical SRP RNAs by exhibiting an elongated apical loop of approximately 10 bp instead of a conserved tetraloop [9] (Fig 1B). To extend and confirm our previous study, which demonstrated binding of cpSRP54 to the cpSRP RNA using anion exchange chromatography [9], complex formations between the SRP components of *Physcomitrella patens* were analyzed using gel filtration chromatography with the recombinant proteins and *in vitro* transcribed cpSRP RNA. Because cpSRP54 and full-length, mature cpSRP43 showed the same running behavior in SDS-PAGE, an N-terminal truncated cpSRP43 construct lacking chromodomain one (Pp-cpSRP43ΔCD1) was used. When a combination of cpSRP54 and cpSRP RNA was loaded onto the column, a large fraction of both components coeluted as a complex with a molecular weight of approximately 170 kDa, while the single components eluted in separate fractions (Fig 5). The binding of the cpSRP RNA to cpSRP54 was specific; no binding between the RNA component and cpSRP43 was observed (Fig 5). The functionality of the recombinant cpSRP43 was controlled by confirming complex formation with cpSRP54 as previously described (Fig 5, [9]). Together, these data confirm the ability of *Physcomitrella patens* cpSRP54 to form a stable complex with the cpSRP RNA.

**Fig 6:**
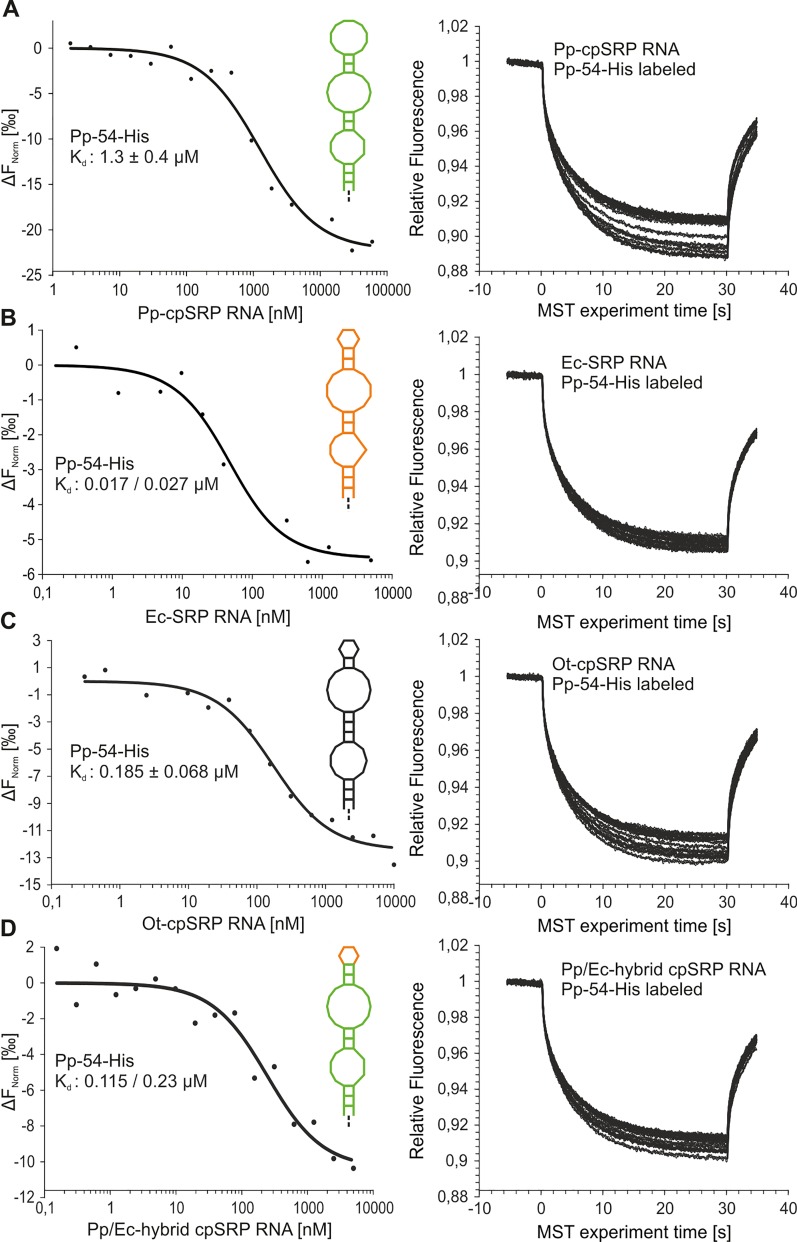
Determination of binding affinities between *Physcomitrella patens* cpSRP54 and various SRP RNAs using microscale thermophoresis. Binding affinities of *Physcomitrella patens* cpSRP54 complex formation with *Physcomitrella patens* cpSRP RNA (A), *E*. *coli* SRP RNA (B), *Ostreococcus tauri* cpSRP RNA (C), and the *Physcomitrella patens/E*. *coli* (Pp/Ec)-hybrid cpSRP RNA (D). The structure of used (cp)SRP RNAs are given in the left panels: (green) *Physcomitrella patens*, (orange) *E*. *coli*, (black) *Ostreococcus tauri* and (orange/green) *Physcomitrella patens/E*. *coli*-hybrid. Fluorescently labeled Pp-cpSRP54 was kept between 10 to 20 nM, while the indicated (cp)SRP RNAs were titrated in a micromolar excess (e.g., 90 μM Pp-cpSRP RNA, 5 μM Ec-SRP RNA, 10 μM Ot-cpSRP RNA and 5 μM Pp/Ec-hybrid cpSRP RNA). The difference in normalized fluorescence [‰] was plotted against the concentration of the SRP RNAs, and the binding affinity (K_d_) was evaluated using the MO.Affinity Analysis Software (NanoTemper Technologies GmbH, Munich, Germany) (left panel) (right panel, raw MST-traces). All experiments were performed at least twice.

The binding between *Physcomitrella patens* cpSRP54 and (cp)SRP RNAs was analyzed quantitatively using microscale thermophoresis (Fig 6A–6D and S2A–S2D Fig). Titration of fluorescently labeled cpSRP54-His with increasing amounts of cpSRP RNA yielded a K_d_ value of 1.3 ± 0.4 μM (Fig 6A and S2A Fig). This indicates a >15.000-fold weaker binding than that observed for the SRP system in *E*. *coli*, exhibiting a K_d_ value in the picomolar range (52 ± 5 pM) [22]. To elucidate whether the fluorescently labeled His-tagged *Physcomitrella patens* cpSRP54 is generally able to interact efficiently with a classical SRP RNA, the interaction with the SRP RNA of *E*. *coli* was analyzed. K_d_ values of 17 nM and 27 nM were determined, reflecting high affinity binding (Fig 6B and S2B Fig). Furthermore, the use of the cpSRP RNA of the unicellular green algae *Ostreococcus tauri*, which belongs to the classical SRP RNAs and is characterized by the conserved GNRA tetraloop in addition to the symmetric and asymmetric internal loops, resulted in a K_d_ value of 0.185 ± 0.068 μM (Fig 6C and S2C Fig). These data demonstrate that the structure of *Physcomitrella patens* cpSRP54 is conducive to efficient binding of classical SRP RNAs. To analyze whether the elongated apical loop of the *Physcomitrella patens* cpSRP RNA causes low affinity binding to cpSRP54, a hybrid SRP RNA was generated by replacing the elongated loop with the tetraloop of *E*. *coli* SRP RNA (Pp/Ec-hybrid cpSRP RNA). K_d_ values of 0.115 μM and 0.23 μM were determined for the interaction with *Physcomitrella patens* cpSRP54 (Fig 6D and S2D Fig), indicating that the hybrid SRP RNA binds to *Physcomitrella patens* cpSRP54 with a 6- to 11-fold stronger affinity than the wildtype cpSRP RNA. Therefore, these data indicate that the unusual elongated apical loop of *Physcomitrella patens* cpSRP RNA contributes to the relatively low affinity cpSRP54/cpSRP RNA complex in *Physcomitrella patens*.

## Discussion

In this study, we analyzed the complex formation between cpSRP protein subunits and cpSRP RNA in *Chaetosphaeridium globosum* and *Physcomitrella patens* to extend our knowledge of the evolution from the prokaryotic to the higher plant chloroplast SRP system. Within the cytosolic SRP system of *E*. *coli*, the SRP RNA has an essential function. It accelerates the Ffh/FtsY complex formation and stimulates the GTPase activation of the two GTPases in this complex [23–25]. The chloroplast SRP system of spermatophytes functions in absence of a SRP RNA and various mechanisms were developed to enable posttranslational LHCP transport without this RNA component. First, the structure of receptor protein cpFtsY differs from bacterial FtsY by a preorganized conformation that enables efficient interaction with cpSRP54 without structural rearrangements [26–29]. Second, the cpSRP54M domain mimics the function of the SRP RNA in that it significantly accelerates the cpSRP54/cpFtsY complex formation [30]. The most striking feature of posttranslational LHCP transport is the existence of the chloroplast-specific cpSRP43 subunit [7, 8]. The cpSRP complex formation is achieved by the interaction of the conserved A(R/K)R binding motif within the C-terminal region of cpSRP54 and the residues forming a twinned aromatic cage within the CD2 of cpSRP43 [15, 18, 19]. Notably, however, recent data demonstrated that the cpSRP43 subunit does not interact with cpSRP54 in chlorophytes, which is mainly caused by an alteration within the cpSRP43 binding motif of cpSRP54 and an interfering proline in cpSRP43-CD2 [15]. These observations raised the questions of when the cpSRP54/cpSRP43 complex evolved and what evolutionary advantage was achieved. To address these questions, we have analyzed the cpSRP system of selected organisms within the streptophytes. We clearly show a cpSRP54-cpSRP43 interaction in the charophyte *Chaetosphaeridium globosum*. However, unlike the *Arabidopsis thaliana* cpSRP, the affinity of the *Chaetosphaeridium globosum* cpSRP complex is weak. It was observed that a mutation of the aromatic tyrosine 269 in the second aromatic cage of cpSRP43 CD2 led to a drastic loss of affinity of cpSRP43 to cpSRP54 in *Arabidopsis thaliana* [19], so we tested whether the negatively charged aspartate at position 189 within cage 2 of *Chaetosphaeridium globosum* cpSRP43 (Fig 1D) interferes with cpSRP54 binding. Therefore, the mutant construct *Chaetosphaeridium globosum* cpSRP43(D189Y) was generated to mimic the *Arabidopsis thaliana* cage 2 region. However, we did not observe an improved affinity in the cpSRP complex formation using cpSRP43(D189Y) and cpSRP54M of *Chaetosphaeridium globosum* (Kd: 10 μM / 11 μM, S3A and S3B Fig), suggesting that additional regions in cpSRP43 might contribute to the formation of the binding interface. During the transition from water to land plants, mosses form the direct descendants of charophytes. One well-studied moss is *Physcomitrella patens*, which belongs to the bryophyte branch. In this study, we show low affinity binding of *Physcomitrella patens* cpSRP54 to cpSRP43. Similar to *Chaetosphaeridium globosum* cpSRP43, aromatic cage 2 of *Physcomitrella patens* cpSRP43 differs from that of *Arabidopsis thaliana* (Fig 1D); it carries a neutral threonine instead of a tyrosine residue, which probably contributes to a weaker affinity binding to cpSRP54. Additionally, we observed low affinity binding between cpSRP54 and the cpSRP RNA of *Physcomitrella patens*. In *E*. *coli* the binding of Ffh to the SRP RNA displays a very high affinity and is mediated by the symmetric and asymmetric internal loops of the SRP RNA and two alpha helices of the FfhM domain [31]. The conserved SRP RNA tetraloop interacts with FtsY to stabilize the early intermediate of the SRP/FtsY complex [32]. Although current data indicate that the apical loop is not directly involved in Ffh binding in the bacterial system, our results suggest that the elongated loop of *Physcomitrella patens* cpSRP RNA interferes with cpSRP54 binding. First, we observed that (cp)SRP RNAs with the conserved tetraloop structure (from *Ostreococcus tauri* and *E*. *coli*) bind to *Physcomitrella patens* cpSRP54 with a much higher affinity (about 10- to 60-fold) than the *Physcomitrella patens* cpSRP RNA, which indicates that *Physcomitrella patens* cpSRP54 is principally able to bind an SRP RNA efficiently. Second, replacement of the elongated loop of *Physcomitrella patens* cpSRP RNA with the apical tetraloop of bacterial SRP RNA led to a 6- to 11-fold increase in binding affinity. Because it is unlikely that the apical loop is directly involved in cpSRP54 binding, we assume that the elongated loop indirectly influences complex formation with cpSRP54. In yeast, it has been shown that the replacement of the wild-type GAAA tetraloop with an UUCG tetraloop strongly reduced the association with SRP54 [33]. Subsequent enzymatic probing experiments demonstrated that tetraloop substitutions not only change the structure of the tetraloop itself but can also influence the conformation of the symmetric internal loop, which contains bases critical for SRP54 binding [34]. The indirect influence of changes in the apical loop on the internal loop is probably due to alterations in the thermodynamic stability of the SRP RNA, which is described as a flexible molecule which can adopt several thermodynamically stable configurations [35, 36]. However, as nothing is known about the structure of cpSRP54/cpSRP RNA complexes in plants it is also possible that the large apical loop of the *Physcomitrella patens* cpSRP RNA might sterically interfere with cpSRP54 binding.

The evolutionary benefit of recruiting cpSRP54 to transit complex formation with LHCP by interaction with cpSRP43 is very likely an improvement of targeting efficiency. Although the analysis of LHCP transport in the *Arabidopsis thaliana* cpSRP pathway mutants demonstrates that cpSRP43 alone is able to transport the LHCPs [37], it was shown that mutations within cpSRP54, which are detrimental to the cpSRP complex formation, showed a reduced LHCP insertion *in vitro* [7, 18]. This can be explained by the recent finding that cpSRP54 modulates the structural dynamics of cpSRP43 and enhances the binding affinity of cpSRP43 to LHCP [38, 39]. Furthermore, recent data suggest that cpSRP54 might play a direct role in LHCP recognition because it was demonstrated that cpSRP54 is required for the formation of a low molecular weight transit complex [15]. In this study we show low affinity binding of cpSRP54 to cpSRP43 and to the cpSRP RNA in *Chaetosphaeridium globosum* and *Physcomitrella patens*. These findings together with the observation of conserved cpSRP54- and cpSRP43-binding motifs within charophytes and the widespread occurrence of cpSRP RNAs with atypical apical loops in charophytes and bryophytes ([9, 15, 17] and this work) indicate that the formation of the heterodimeric cpSRP complex has developed within the charophytes and that the cpSRP systems of charophytes and bryophytes represent evolutionary intermediates in the evolution of the high affinity posttranslational cpSRP complex, and the complete loss of the SRP RNA component. However, it remains unclear which role the cpSRP RNA in charophytes and bryophytes might play, and how higher plants compensate for the loss of the cpSRP RNA in cotranslational protein transport.

## Supporting Information

S1 Fig Supplemental MST-measurements to Fig 4 (left panel, evaluated affinity; right panel, raw MST traces). (TIF)

S2 Fig Supplemental MST-measurements to Fig 6 (left panel, evaluated affinity; right panel, raw MST traces). (TIF)

S3 Fig Determination of the binding affinity of *Chaetosphaeridium globosum* cpSRP54M/cpSRP43D189Y complex formation using microscale thermophoresis (left panel, evaluated affinity; right panel, raw MST traces). (TIF)

S1 Table Amino acid sequence of *Chaetosphaeridium globosum* cpSRP54M and cpSRP43 corresponding to materials and methods ´*Plasmids and plasmid construction´*. (PDF)
