# Supplementary figures and images for: The Chloroplast SRP Systems of Chaetosphaeridium globosum and Physcomitrella patens as Intermediates in the Evolution of SRP-Dependent Protein Transport in Higher Plants
Source: PLoS One. 2016 Nov 18;11(11):e0166818. doi: 10.1371/journal.pone.0166818 (PMC5115805; doi:10.1371/journal.pone.0166818)

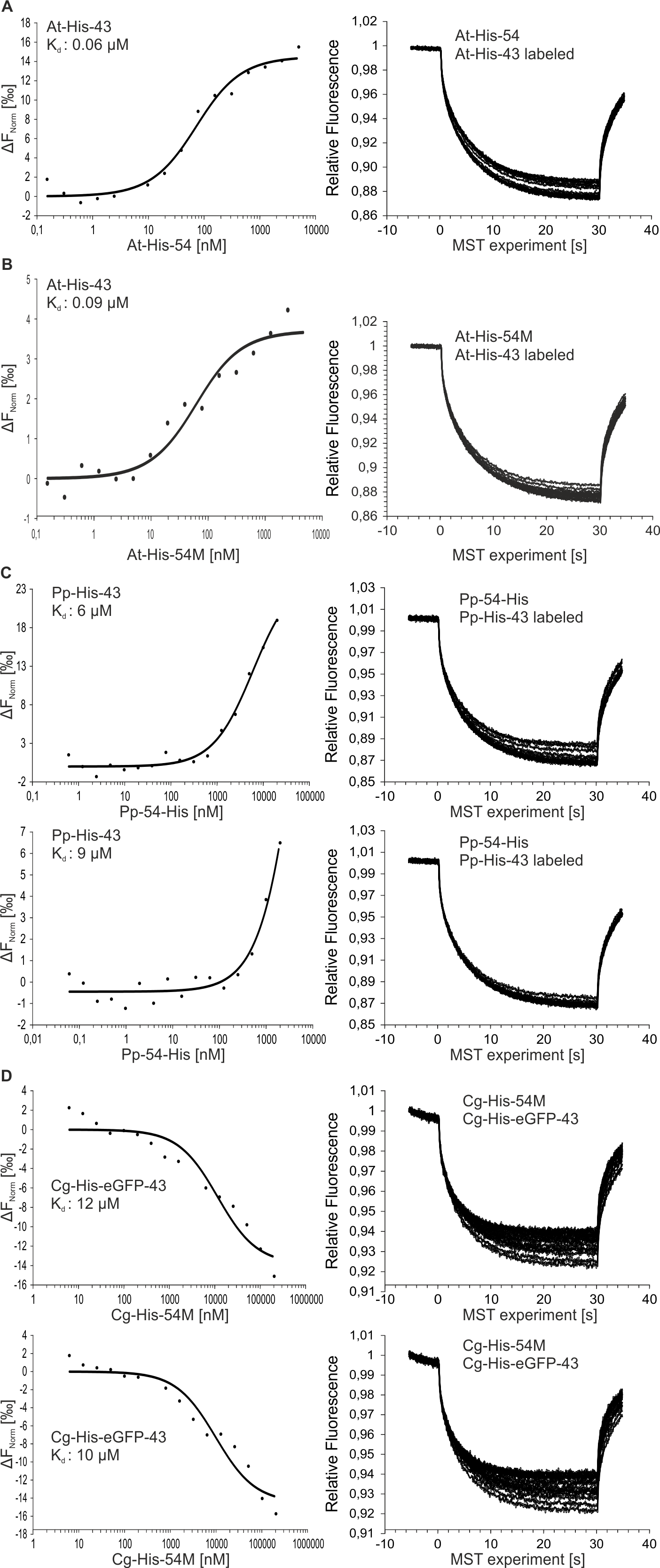

Supplement: S1 Fig — (TIF) [file pone.0166818.s001.tif]

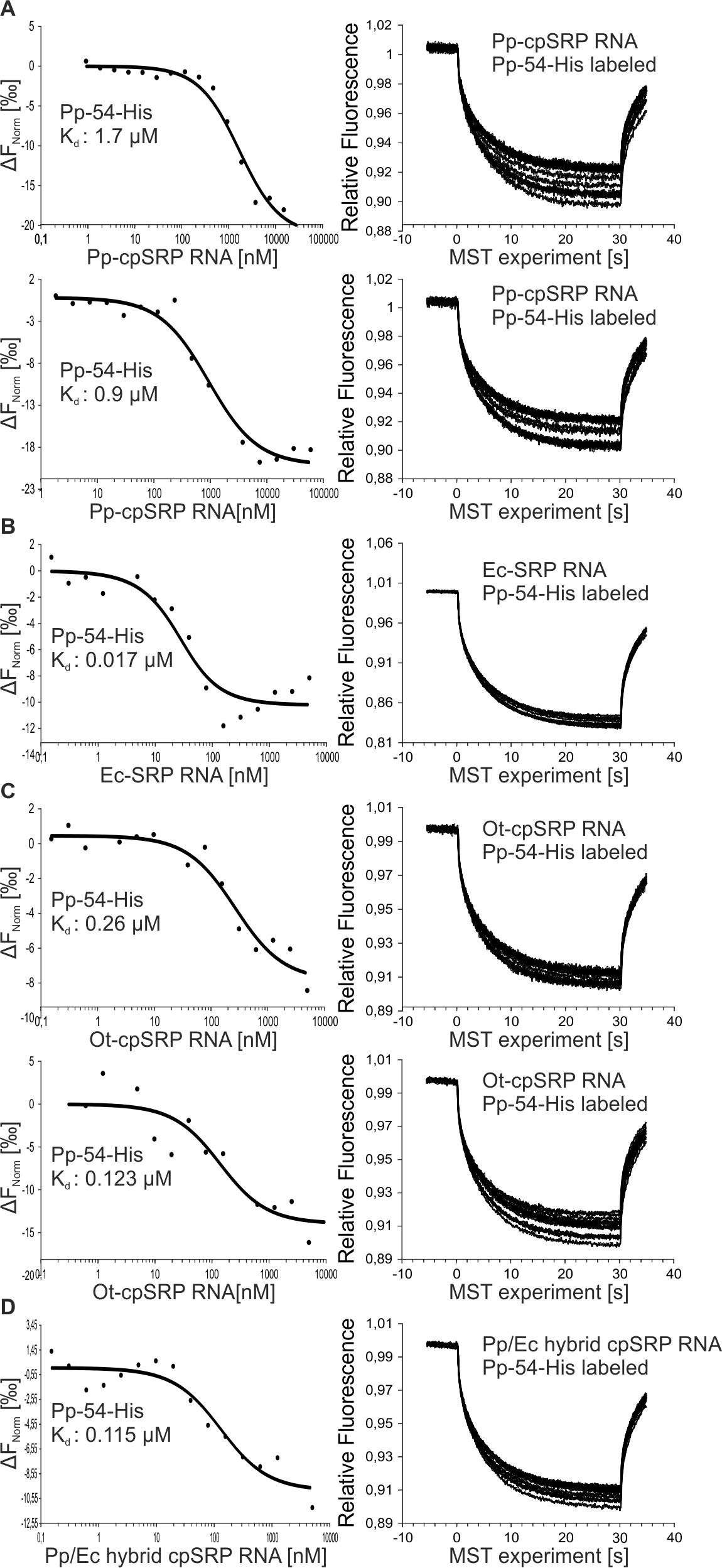

Supplement: S2 Fig — (TIF) [file pone.0166818.s002.tif]

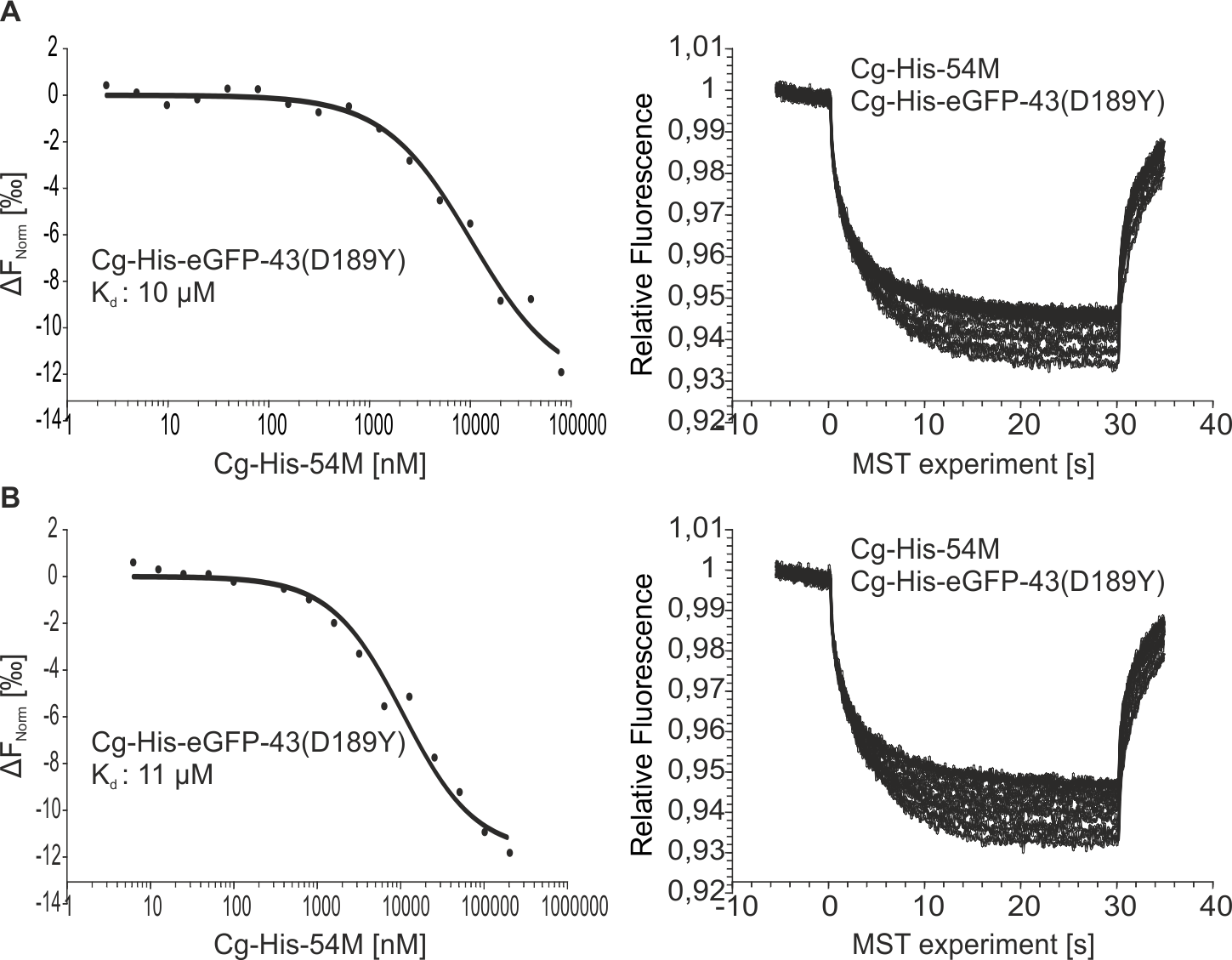

Supplement: S3 Fig — (TIF) [file pone.0166818.s003.tif]
