# Supplementary material for: The Chloroplast SRP Systems of Chaetosphaeridium globosum and Physcomitrella patens as Intermediates in the Evolution of SRP-Dependent Protein Transport in Higher Plants
Source: PLoS One. 2016 Nov 18;11(11):e0166818. doi: 10.1371/journal.pone.0166818 (PMC5115805; doi:10.1371/journal.pone.0166818)
Supplement: S1 Table — (PDF) [file pone.0166818.s004.pdf]

Organism/Protein	Amino acid sequence
Chaetosphaeridium globosum	
cpSRP54M	MASRILGMGDVVSFVEKAQELMEEEE<br>AKVIQEKIRTAKFDFDDMLKQTQMVV<br>KMGNMSGMMKMMPGANKVTPQQIY<br>EAEKAMKMMEAMIQVMEPEERKDPE<br>LLAKSPSRRRRIAEASGYEPEQVSQIVQ<br>QLFTMRAKMKNIMGGIPGVGDDPLRG<br>QKKAAPGTAKRKKQLAGRAPAAGFG<br>AKKG
cpSRP43	MEWEVEKIVSSRIIDRQTQYLVRWAD<br>DHPDSWEPGENIAGDLVTDYESAWW<br>QAARKADEAKLKELLDANELRDVNAI<br>DENQRTAVLFAAGLGNDSCVKMLIEN<br>GADIAWEDKDGYTPLHIAAGYVHLQV<br>VQALLAAGADPEATDKQGRSPLELSQ<br>QLLERAPRMNPLQFARRMALDQVVK<br>ELDAAVFEDVGVSELLDKRKNAGGK<br>WEYLVRWSDDVEDSWVPEGAVDEQII<br>KDFNAGLEYGIAEKVLEKRDVENVGA<br>EYLVKWADLDEPTWEPELNVAPEVIA<br>EFENVPVEEVEERQHLLQRKEEAALE<br>AAQAAAKEALVVNANAPTTSS
